# Four factors as key performance indicators of game outcomes in the Women’s U19 World Cup and Women’s EuroBasket 2025

**DOI:** 10.1186/s13102-026-01720-x

**Published:** 2026-04-29

**Authors:** Ľubor Tománek, Štefan Suja, Rocco Di Michele, Feng Li, Tomáš Vencúrik

**Affiliations:** 1 Department of Sports Games, Faculty of Physical Education and Sport, Comenius University Bratislava, Bratislava, Slovakia; 2 Department for Life Quality Studies, University of Bologna, Bologna, Italy; 3 International Joint Laboratory on High Performance Sports Research, Beijing Sport University, Beijing, China; 4 Research Center for Basketball Performance, Beijing Sport University, Beijing, China; 5 Department of Sport Performance and Exercise Testing, Faculty of Sports Studies, Masaryk University, Kamenice 5, Brno, 62 500 Czechia

**Keywords:** Performance Indicators, Team Success Predictors, Efficiency Metrics, International Competitions

## Abstract

**Background:**

Among the most influential statistical metrics in basketball is Oliver’s Four Factors model, which includes effective field goal percentage (eFG%), turnover percentage (TOV%), offensive rebound percentage (ORB%), and free-throw rate (FTR). Although widely used in men’s basketball, its application in women’s competitions, particularly at youth levels, remains limited. This study examined the predictive value of Oliver’s Four Factors (eFG%, TOV%, ORB%, FTR) for game outcomes in women’s basketball at the FIBA U19 Women’s Basketball World Cup 2025 (WCU19w 2025) and Women’s EuroBasket 2025 (EBw 2025).

**Methods:**

A total of 85 games (170 team performances) were analysed across both tournaments. Game-related statistics were extracted from official FIBA sources and used to compute the Four Factors. Differences between winning and defeated teams were assessed, and binary logistic regression was applied to identify key predictors.

**Results:**

Winning teams at the WCU19w 2025 showed higher eFG%, ORB%, and lower TOV% (*p* < .05), with eFG% and ORB% identified as significant predictors. At the EBw 2025, significant differences were found in eFG%, TOV%, and FTR (*p* < .05), with eFG% as the strongest predictor, followed by TOV% and FTR.

**Conclusions:**

Overall, eFG% was the most consistent determinant of success across both competitions. However, performance indicators varied by category: offensive rebounding was more relevant in U19, while free-throw rate and turnover control were more important in senior teams. These findings support the applicability of the Four Factors model in women’s basketball and highlight the need to adjust performance priorities according to competitive level.

## Background

Research in basketball performance over the past decades has focused on identifying game indicators that best distinguish winning teams from losing ones. The most frequently mentioned factors include shooting efficiency, rebounds, and assists, which have a decisive impact on game outcomes [1, 2]. In women’s basketball, winning teams have been shown to achieve significantly higher two-point shooting accuracy, more defensive rebounds, and more assists, as confirmed by an analysis of games from the Tokyo 2020 Olympic Games [3]. Similar findings have been reported by other studies, highlighting that shooting efficiency and rebound value are among the main determinants of team success [4, 5].

Studies also point to regional differences in the playing style of women’s teams. European championships are characterized by more organized offense and a slower game pace, whereas African teams exhibit a higher number of free throws and turnovers [6]. Research further confirms the importance of foreign players in national club competitions, who generally achieve better offensive statistics, while domestic players excel in defense [7, 8].

Modern basketball, including the women’s game, is strongly influenced by the growing role of three-point shooting, which alters game dynamics and enhances offensive efficiency [9–11]. Studies indicate that a higher three-point shooting percentage is associated with better team performance [5, 12], although its success depends on game context and the specific characteristics of women’s basketball [13]. In this context, the increasing frequency of three-point attempts has emerged as a key tactical element of modern basketball, fundamentally reshaping offensive strategies and overall game dynamics [8, 10, 14, 15]. Higher three-point shooting accuracy is consistently linked to team success [16, 17].

Key performance indicators also include shooting accuracy, rebounds, and assists [1, 2, 18]. In women’s basketball, winning teams consistently demonstrate higher success rates in two-point and three-point shots, more defensive rebounds, and a greater number of assists [3, 19]. Shooting efficiency, or free-throw percentage, remains one of the most significant factors influencing game outcomes [14, 18]. The importance of these factors has been confirmed across various contexts—from the Olympic Games [3] and European or African championships [11], through professional leagues such as EuroLeague Women [6, 13, 19], to the WNBA, where shooting efficiency accounts for approximately 42% of the impact on game results [20]. Systematic evaluation of game performance plays a crucial role in assessing team effectiveness, planning tactics, and advancing the development of women’s basketball [4, 9]. These insights confirm that performance in women’s basketball results from a complex interaction of technical-tactical, physical, and psychosocial factors. Analytical approaches, therefore, represent an indispensable tool for coaches and analysts seeking effective strategies to enhance team success [9].

Oliver (2004) [21] identified Four Factors that decisively influence NBA game outcomes. Each of these factors is directly associated with the termination of a team’s possession in offensive phase of the game: shooting efficiency, typically expressed as effective field goal percentage (eFG%), turnover percentage (TOV%), offensive rebounding percentage (ORB%), and free throw rate (FTR). While possessions may also end due to contextual constraints, such as the end of a quarter or foul-related stoppages, such events occur infrequently and have a minimal impact on season-long possession data. Therefore, performance analysis should primarily focus on the four dominant possession-ending outcomes when evaluating offensive efficiency [22]. Although possession-based models focusing on dominant possession-ending outcomes have been increasingly adopted in men’s basketball research, their application in women’s basketball remains limited. This is particularly evident at the youth level, where performance analysis has traditionally relied on aggregated statistics rather than possession-level indicators. Consequently, the lack of empirical evidence using this model in female youth basketball represents a methodological gap in the literature, highlighting the need for research that accounts for the specific developmental, tactical, and physiological characteristics of young female players.

The aim of this study was to identify differences between winning and defeated teams in Oliver’s Four Factors (eFG%, TOV%, ORB%, FTR) and to determine the extent to which these factors predict game outcomes in women’s basketball. Specifically, the study analyzed teams’ performances at the FIBA U19 Women’s Basketball World Cup 2025 (WCU19w 2025) and the Women’s EuroBasket 2025 (EBw 2025) to identify the factors that most significantly predict winning outcomes across youth and senior competitions. It was hypothesized that significant differences would exist in the key performance factors influencing game outcomes between EBw 2025 and WCU19w 2025, reflecting variations in performance determinants between senior and youth competitions. The findings aim to provide insights for coaches and analysts on key performance indicators that influence game outcomes.

## Methods

### Research sample

The sample consisted of team-game performances obtained from two major women’s international competitions, the WCU19w 2025 and EBw 2025. The WCU19w 2025 featured 15 teams, with a total of 49 games—21 in the group stage and 28 in the knockout and placement rounds. The EBw 2025 featured 16 teams and 36 games—24 in the preliminary round and 12 in the knockout stage. In total, 85 games across both tournaments were analyzed.

### Research design

The present study is grounded in an observational methodology, commonly used in sports performance analysis to examine real-game behavior without experimental manipulation [23]. A total of 85 games across both tournaments were included in the analysis, yielding 170 team-game performances. Each game contributed two team-level performances (one per team), which were treated as independent units of analysis, as all performance indicators were computed separately for each team and represented team-specific outcomes. Selected team performance variables (field goals attempts – FGA, field goals made – FGM, three-point made – 3PM, free throws attempted – FTA, free throws made – FTM, offensive rebounds – ORB, defensive rebounds – DRB, turnovers – TOV) were extracted from official FIBA box scores and subsequently used to calculate Oliver’s Four Factors: eFG%, TOV%, ORB%, and FTR. All performance indicators in box scores from the games were recorded by specially trained statisticians from the FIBA organization, who obtain an international-level license upon completing their training. Individual player performance, as well as the involvement or contribution of individual players to overall team performance, was not examined.

### Procedures

For each game, we monitored the parameters listed in Table 1, which were necessary to identify and calculate Oliver’s Four Factors [21, 22]: eFG%, TOV%, ORB%, and FTR. Statistical data were obtained by analyzing game-related statistics (official box scores) from individual games played at WCU19w 2025 (https://www.fiba.basketball/en/events/fiba-u19-womens-basketball-world-cup-2025/games) and EBw 2025 (https://www.fiba.basketball/en/events/fiba-womens-eurobasket-2025/games), sourced from the official FIBA websites.

**Table 1:** Total number of monitored indicators at WCU19w 2025 and EBw 2025

Indicator	WCU19w 2025 (*n* = 49)		EBw 2025 (*n* = 36)	
	Winners	Defeated	Winners	Defeated
FGA	3,366	3,038	2,169	2,196
FGM	1,423	1,023	1,016	826
3PM	401	290	276	217
FTA	1,079	905	726	613
FTM	777	606	578	430
ORB	799	559	357	384
DRB	1,549	1,246	1013	847
TOV	773	921	473	536

*FGA* field goals attempted, *FGM* field goals made, *3PM* three-point made, *FTA* free throws attempted, *FTM* free throws made, *ORB* offensive rebounds, *DRB* defensive rebounds, *TOV* turnovers

Oliver’s Four Factors were calculated using the following formulas:

$$\:eFG\%=\:\frac{(FGM+0.5*3PM)}{FGA}$$

Note: eFG% – effective field goal percentage; FGM – field goals made; 3PM – three-point made; FGA – field goal attempted

$$\:TOV\%=\:\frac{TOV}{(FGA+0.44*FTA+TOV)}$$

Note: TOV% – turnover percentage; FGA – field goals percentage; FTA – free throws attempted

$$\:ORB\%=\:\frac{ORB}{\left(ORB+Opp\:DRB\right)}$$

Note: ORB% – offensive rebound percentage; ORB – offensive rebounds; Opp DRB – opponent defensive rebounds

$$\:FTR=\:\frac{FTM}{FGA}$$

Note: FTR – free throw rate; FTM – free throws made; FGA – field goals attempted.

### Statistical analysis

Data are presented as mean ± standard deviation. The normality of data distribution was verified using the Shapiro–Wilk test. The homogeneity of variance was tested by Levene’s test. For data meeting the assumption of normality, differences were evaluated using an independent samples t-test. For variables not meeting this assumption of normality, the non-parametric Mann–Whitney U test was applied. If the assumption of homogeneity of variance was violated, the Welch’s test was used. All tests were selected as they are appropriate for comparing two independent groups (winning vs. defeated teams). Each team’s performance indicators were calculated separately and treated as independent observations [24, 25]. Practical significance was interpreted using Cohen’s d, with thresholds of 0.2 for a small effect, 0.5 for a medium effect, 0.8 for a large effect, and 1.3 for a very large effect [26]. Binary logistic regression (enter method) was used to predict game outcomes based on the following independent variables: eFG%, TOV%, ORB%, and FTR [27]. Game outcomes were coded dichotomously as 0 (loss) or 1 (win). Regression coefficients were estimated using the maximum likelihood method, and their statistical significance was evaluated using Wald’s test. In addition, 95% confidence intervals were calculated for the odds ratios. The Nagelkerke R² statistic was employed to determine the proportion of variance in the dependent variable (game outcome) explained by the predictors (eFG%, TOV%, ORB%, and FTR). The significance level was set at *p* < .05. All statistical analyses were performed using IBM SPSS Statistics, version 29 (IBM Corp., Armonk, NY, USA).

## Results

Descriptive characteristics, as well as the statistical significance between winning and defeated teams in eFG%, TOV%, ORB%, and FTR across the respective tournaments, are presented in Table 2. At WCU19w 2025, we identified statistically significant differences between winning and defeated teams in almost all parameters, with medium to large effect sizes. For FTR, no statistically significant differences were observed, with a small effect size. At EBw 2025, statistically significant differences were found only in two indicators: eFG% and FTR, with large and medium effect sizes, respectively. For ORB% and TOV%, no statistically significant differences were detected between winning and defeated teams, with small effect sizes.

**Table 2:** Descriptive characteristics (mean ± standard deviation), statistical significance, and effect sizes at WCU19w 2025 and at EBw 2025

Indicator	Winners	Defeated	*p*	ES (95% CI)
WCU19w 2025				
eFG%	48.1 ± 8.6	38.5 ± 8.1	< 0.001	1.15 (0.72; 1.58)
TOV%	16.9 ± 4.2	21.2 ± 7.0	< 0.001	0.74 (0.33; 1.15)
ORB%	390 ± 11.8	25.7 ± 9.37	< 0.001	1.25 (0.82; 1.68)
FTR	23.5 ± 10.2	20.8 ± 12.3	0.11	0.25 (-0.15; 0.64)
EBw 2025				
eFG%	53.4 ± 8.1	42.6 ± 6.9	< 0.001	1.44 (0.91; 1.95)
TOV%	16.0 ± 4.3	17.9 ± 5.8	0.12	0.37 (-0.1; 0.83)
ORB%	29.9 ± 8.7	27.3 ± 9.8	0.24	0.29 (-0.2; 0.75)
FTR	27.1 ± 11.6	20.5 ± 11.6	0.02	0.57 (0.09; 1.04)

*eFG%* effective field goal percentage, *TOV%* turnover percentage, *ORB%* offensive rebound percentage, *FTR* free throw rate, *p* level of statistical significance, *ES* effect size, *CI* confidence interval

Analysis of data from WCU19w 2025 confirmed that eFG% and ORB% were significant predictors of victory, whereas TOV% and FTR were not (Table 3). Concerning eFG%, an increase of one-percent increase in shooting efficiency raised the odds of winning by approximately 16%. Also, a one-unit increase in ORB% raised the odds of winning by 14.5%. The Nagelkerke R² value of 0.61 indicates that the predictor variables explain approximately 61% of the variance in the dependent variable. TOV% and FTR were not significant predictors of team success in this age category.

The results of the binary logistic regression indicated that eFG%, TOV%, and FTR were significant predictors of victory at EBw 2025 (Table 3). An increase of one unit in eFG% and FTR increases the odds of winning by 40.1% and 7.9%, respectively. Conversely, a one-unit increase in TOV% decreased the odds of winning by 28.5%. The ORB% indicator was not statistically significant, although the coefficient’s direction suggested a positive trend. At this event, the Nagelkerke R² value of 0.67 indicates that the predictor variables explained approximately 67% of the variance in the dependent variable.

**Table 3:** Estimation of regression model parameters for winning at WCU19w 2025 and EBw 2025

Variable	B	SE	Wald	df	*p*	OR	95% CI for OR	
							Lower	Upper
WCU19w 2025								
eFG%	0.147	0.039	14.175	1	< 0.001	1.158	1.073	1.251
TOV%	− 0.097	0.06	2.557	1	0.11	0.908	0.807	1.022
ORB%	0.135	0.034	15.496	1	< 0.001	1.145	1.07	1.224
FTR	0.036	0.027	1.788	1	0.181	1.036	0.984	1.092
EBw 2025								
eFG%	0.337	0.089	14.383	1	< 0.001	1.401	1.177	1.667
TOV%	− 0.335	0.117	8.239	1	0.004	0.715	0.569	0.899
ORB%	0.037	0.047	0.599	1	0.439	1.037	0.945	1.138
FTR	0.076	0.035	4.711	1	0.030	1.079	1.007	1.156

*eFG%* effective field goal percentage, *TOV%* turnover percentage, *ORB%* offensive rebound percentage, *FTR free throw rate*, *B* standardized beta weights, *SE* standard error of the estimate, *Wald* values of Wald’s test, *df* degrees of freedom, *p* the statistical significance of regression coefficients, *OR* odds ratio, *CI* confidence interval

## Discussion

The present study aimed to identify differences between winning and defeated teams in Oliver’s Four Factors (eFG%, TOV%, ORB%, FTR) and to determine the extent to which these factors predict game outcomes in women’s basketball. The four-factor model [21] provides a comprehensive framework for understanding basketball performance, and the results of this study largely support its structure. At WCU19w 2025, winning teams achieved a 9.6% higher eFG% than defeated teams (*p* < .001, d = 1.15), underscoring the dominant influence of shooting efficiency. A substantial 4.3% lower TOV% among winners (*p* < .001, d = 0.74) further highlights the importance of possession management. Similarly, the 13.3% higher ORB% of winning teams (*p* < .001, d = 1.25) confirms the relevance of second-chance scoring, while the smaller 2.7% difference in FTR still favored successful teams. At EBw 2025, comparable trends were observed: winners achieved a 10.8% advantage in eFG% (*p* < .001, d = 1.44), recorded 1.9% fewer turnovers, and posted a small ORB% difference of 2.6%, likely reflecting greater balance among senior teams and stronger defensive organization. Notably, the 6.6% higher FTR among winning teams (*p* < .02, d = 0.57) emphasized a more aggressive, contact-oriented offensive approach in senior competition.

The results of the binary logistic regression from WCU19w 2025 and EBw 2025 clearly indicate that shooting efficiency, as measured by eFG%, was the most consistent and influential predictor of success across both tournaments. As hypothesized, the comparison of WCU19w 2025 and EBw 2025 reveals meaningful differences between youth and senior competitions. Among U19 teams, eFG% and ORB% were the dominant performance factors, whereas in senior teams the emphasis shifted toward shooting efficiency combined with the ability to draw free throws (FTR). This shift may reflect the influence of accumulated game intelligence, tactical discipline, greater playing experience, and differences in playing slyle, as well as higher levels of physical preparedness [9, 28]. At the senior level (EBw 2025), turnovers (TOV%) also played a significant role, while at WCU19w 2025, offensive rebounding (ORB%) emerged as a key contributing factor. These findings align with previous research identifying shooting efficiency, turnover control, and second-chance opportunities as critical determinants of performance in women’s basketball [1, 29].

Beyond eFG%, the ability to draw fouls (FTR) proved particularly relevant in senior competitions, suggesting that scoring efficiency is shaped not only by shooting accuracy but also by the team’s capacity to generate additional scoring opportunities under defensive pressure [16, 30, 31]. Whereas winners at WCU19w 2025 relied heavily on ORB% to create second-chance scoring situations, the reduced ORB% differences at EBw 2025 may reflect the distinct tactical characteristics of senior teams, in which game experience, physical maturity, and turnover control become more important [1, 6].

Turnovers (TOV%) emerged as a particularly important negative predictor of success at EBw 2025, aligning with previous evidence that emphasizes the role of ball control in maintaining game tempo and offensive efficiency [29]. Minimizing turnovers is especially critical in tightly contested games, where small differences in possession quality can determine the final result. The combined influence of high eFG% and effective foul drawing (FTR) further demonstrates that maximizing scoring efficiency while minimizing ball turnover directly increases the likelihood of victory [15]. At EBw 2025, TOV% emerged as a direct determinant of winning probability. Interesting trends were observed in previous analyses of women’s continental championships, where performance varied across regions—Asian teams excelled in shooting efficiency, while African teams recorded higher turnover rates, reflecting differences in technical development, tactical culture, and coaching approaches [11, 18]. These findings reinforce the idea that team success depends not only on individual performance but also on contextual factors such as regional playing styles and the strategic orientation of coaching staff.

Several limitations should be considered when interpreting the findings. The analysis drew on data from two tournaments (WCU19w 2025 and EBw 2025), which may limit the extent to which the results can be generalized to other competitions or age categories. While the dataset was sufficient to address the study objectives, a larger sample could further strengthen the robustness of the conclusions. In addition, the analytical framework was centered on Oliver’s Four Factors, and the inclusion of additional advanced performance indicators may provide complementary insights in future research. Finally, the unavailability of final team rankings and the use of categorized score differences may have slightly constrained the precision of certain interpretations.

Future research should therefore expand the dataset to include games from multiple tournaments and various age categories to increase the robustness and generalizability and enhance external validity. Incorporating additional advanced performance indicators, considering final team rankings, and analyzing score-difference values would provide a more nuanced understanding of the factors that influence game outcomes across different levels of competitiveness. Furthermore, within this framework, future studies should also address regional differences at the World Championship level to provide deeper insight into potential variations in performance across regions. Although the four-factor model has been shown to reliably predict team success, it is inherently possession-based. However, a comprehensive evaluation of team performance also requires consideration of defensive performance indicators, which are not explicitly accounted for in the four-factor framework.

## Conclusions

The application of Oliver’s Four Factors framework is more commonly observed in season-long analyses of professional basketball leagues than in short-term international tournaments such as the World Cup and the European Championship. Furthermore, its use is more prevalent in men’s basketball than in women’s or youth basketball. Overall, our findings confirm that eFG% remains a consistent predictor of victory across age categories and tournaments, whereas complementary indicators, such as TOV% and ORB%, vary by age category and competition level. Differences between age categories suggest that younger teams benefit primarily from high eFG% and ORB%. In contrast, senior players rely more on free-throw accuracy and turnover control. This shift likely reflects greater player experience, improved decision-making quality, enhanced physical preparedness, and refined tactical intelligence.

The results underscore the importance of an analytical approach in evaluating women’s basketball teams’ performance. Key determinants of success include shooting efficiency, offensive rebounding, turnover reduction, and free-throw drawing, with priority indicators tailored to the age category and level of competition. Monitoring current developmental trends within a given category is essential, as it provides guidance for coaches not only in the training process but also in the analysis and evaluation of team performance, and the preparation of game strategies for specific tournaments, particularly in the context of national teams. Our study indicates that coaches should focus on the individual components of the Four Factors within the training process, rather than treating them as a single, unified construct.

## Data Availability

The datasets used and/or analysed during the current study are available from the corresponding author on reasonable request.

## Abbreviations

WCU19w 2025: U19 Women’s Basketball World Cup 2025
EBw 2025: Women’s EuroBasket 2025
FGA: field goal attempted
FGM: field goal made
3PM: three–point made
FTA: free throw attempted
FTM: free throw made
ORB: offensive rebound
DRB: defensive rebound
TO: turnover
eFG%: effective field goal percentage
TOV%: turnover percentage
ORB%: offensive rebound percentage
FTR: free throw rate
B: standardized beta weights
SE: standard error of the estimate
Wald: values of Wald’s testd
f: degrees of freedom
p: level of statistical significance
OR: odds ratio
CI: confidence interval
